# Malignant Peritoneal Mesothelioma: An In-Depth and Up-to-Date Review of Pathogenesis, Diagnosis, Management and Future Directions

**DOI:** 10.3390/cancers15194704

**Published:** 2023-09-25

**Authors:** Josh B. Karpes, Raphael Shamavonian, Suzannah Dewhurst, Ernest Cheng, Ru Wijayawardana, Nima Ahmadi, David L. Morris

**Affiliations:** 1 Hepatobiliary and Surgical Oncology Unit, Department of Surgery, St George Hospital, Kogarah, NSW 2217, Australia; 2 St George and Sutherland Clinical School, University of New South Wales, Sydney, NSW 2217, Australia

**Keywords:** malignant peritoneal mesothelioma (MPM), cytoreductive surgery, heated intraperitoneal chemotherapy (HIPEC)

## Abstract

**Simple Summary:**

Mesothelioma is a malignancy of mesothelial tissues. The majority arise from the pleura, but approximately thirty percent of cases arise from the peritoneum. Malignant peritoneal mesothelioma was previously a largely unknown disease, and the extremely poor survival rate reflected that. However, more recently, our understanding has improved, and so has survival. There is, however, an urgent need to investigate further to better understand the disease process, tumour biology, and molecular pathogenesis to establish gold-standard therapeutic strategies. This is, therefore, an in-depth review of the current literature reporting malignant peritoneal mesothelioma with the objective of establishing areas for future research.

**Abstract:**

Malignant peritoneal mesothelioma (MPM) is an extremely rare malignancy usually confined to the abdominal cavity. With an aggressive natural history, morbidity and mortality are consequences of progressive locoregional effects within the peritoneal cavity. The first reported case was in the early 20th century, however, due to the rare nature of the disease and a large gap in understanding of the clinicopathological effects, the next reported MPM cases were only published half a decade later. Since then, there has been exponential growth in our understanding of the disease, however, there are no prospective data and a paucity of literature regarding management. Traditionally, patients were treated with systemic therapy and the outcomes were very poor, with a median survival of less than one year. However, with the advent of cytoreductive surgery and locoregional chemotherapy, there have been significant improvements in survival. Even more recently, with an improved understanding of the molecular pathogenesis of MPM, there have been reports of improved outcomes with novel therapies. Given the disastrous natural history of MPM, the limited data, and the lack of universal treatment guidelines, an in-depth review of the past, present, and future of MPM is critical to improve treatment regimens and, subsequently, patient outcomes.

## 1. Introduction

Mesothelioma is a primary neoplasm of mesothelial cells that line either pleura, peritoneum, or pericardium. The most common type is pleural mesothelioma, which is associated with asbestos, and most knowledge of diagnosis and management arises from experience with this disease [1]. Malignant peritoneal mesothelioma (MPM), a primary tumour of the intra-abdominal serosal membranes, is extremely rare. In the past, understanding of tumour pathogenesis and biology, as well as the natural history of the disease, was scarce and, consequently, treatment options have been limited. However, in recent decades, there has been exponential growth in knowledge of the disease process and its impact. Subsequently, treatment options have improved along with survival outcomes.

## 2. History

The first report on MPM in the literature was in 1908 by Miller and Wynn [2]. The authors described a young adult patient who initially presented with abdominal pain and distension. It was later discovered that his symptoms were attributable to an intra-abdominal malignancy, and within twelve months of diagnosis, the patient was deceased [2]. Later, in 1960, Winslow and Taylor described concerns regarding earlier reports of MPM in the literature based largely on three main issues [3]. Firstly, the authors disputed the validity of post-mortem microscopic examinations, suggesting that metastases from missed small primary tumours may have been misdiagnosed. Secondly, based on tissue sections stained with mucicarmine, they raised the possibility that diagnoses of peritoneal mesothelioma may have been inaccurate. The specimens confirmed the presence of intra-cytoplasmic carminophilic droplets, representing true epithelial mucin and, therefore, indicating a diagnosis of metastatic adenocarcinoma rather than mesothelioma. Finally, a disparity between the clinical picture of the patients who were reported to have MPM, and the morphological behaviour of their pathological autopsy studies may have consequently misrepresented the number of cases that were diagnosed as MPM. After considering these three concerns and re-evaluating the previous case reports of MPM, Winslow and Taylor performed their own clinicopathological analysis of twelve separate cases of MPM, concluding with the first documented suggestion of diagnostic criteria in the literature [3].

Following this, the largest study reported in the literature was a review of 25 patients from a single institution spanning over 30 years from 1950 to 1980 [4]. The authors in this study reiterated previous findings of MPM characterised as an aggressive locally advanced intra-abdominal tumour, which rarely metastasised beyond the peritoneal cavity. Importantly, the authors identified that at the time, treatment options were severely limited, with only one out of the 25 patients partially responding to combined systemic chemo- and radio-therapy, and, therefore, median survival did not exceed ten to twelve months [4].

The following two decades saw a positive shift in outcomes after the introduction of emerging curative treatment options. The combination of cytoreductive surgery (CRS) and chemotherapy localised to the abdominal cavity evolved, and overall survival significantly improved [5,6]. Consensus meetings in 2004 and 2006 proposed this treatment method as the new standard of care for MPM in appropriately selected patients [7,8]. Later, a large systematic review and meta-analysis in 2015 concluded that despite major gaps in the literature owing to the lack of clinical trials, the improvement in survival reported over the recent decades highlighted the efficacy of combination therapy when performed at experienced centers [1]. The authors advocated for a more standardised approach to data classification in the future to establish a gold standard of treatment.

The importance of a standardised approach to MPM was acknowledged by the Peritoneal Surface Oncology Group International (PSOGI), and an expert committee was established to summarise the available data in the literature and select a team of panellists to outline an up-to-date and easily reproducible approach to the work up, management, and follow-up for MPM. The outcome was a panel of 39 specialists made up of mainly surgeons treating rare peritoneal malignancies who established clinical practice guidelines for MPM. The results of their consensus meeting were presented at the PSOGI 2018 international meeting [9].

Around the same time period, two papers in 2016 and 2018 outlined up-to-date reviews on the management of MPM and improved survival with cytoreductive surgery and heated intra-abdominal chemotherapy [10,11]. Sugarbaker et al. suggested that despite promising data on the efficacy of cytoreductive surgery and locoregional abdominal chemotherapy, an alarmingly large proportion of patients with MPM worldwide were only being treated palliatively with systemic chemotherapy [10,11]. The authors evidenced this by referencing Miura et al. [12] (p. 3952), who identified that approximately sixty percent of 1591 patients with MPM between 1973 and 2010 did not receive surgery.

## 3. Objective

With updated evidence in the literature demonstrating a significant survival benefit with operative management in selected patients with MPM, as well as new emerging avenues of immunological and targeted therapy, it is pertinent to provide an up-to-date and in-depth review of MPM. In this paper, we aim to review and evaluate the origin of MPM in the literature, its epidemiology and pathogenesis, the current approach to diagnosis and management, as well as the future direction of therapy. Our objective is to ensure that the rate of failure to treat this potentially curable disease diminishes over the coming years.

## 4. Methodology

This article is a comprehensive and in-depth review of the published literature focusing on the history of MPM and its epidemiology, pathogenesis, diagnosis, management, and the future direction of therapy. We searched the following databases with an unrestricted year of publication: PubMed, Google Scholar and Scopus. The keywords used in the search were “malignant peritoneal mesothelioma”, “primary peritoneal mesothelioma”, “pathophysiology malignant peritoneal mesothelioma”, “review malignant peritoneal mesothelioma” and “management malignant peritoneal mesothelioma”. Only studies written in English were included. After performing the search, there were more than 135 studies, reports and reviews that were deemed appropriate for the context, with 60 of these selected for further examination and use in this updated review. For additional studies, reference lists of relevant research papers were evaluated.

## 5. Epidemiology

MPM constitutes a rare primary intra-abdominal malignancy, with a recent population-based study from the Surveillance Epidemiology and End Results (SEER) database reporting approximately 2000 cases from 1975 to 2016 [13]. Despite these reported numbers, other data suggest there are more than 2500 new cases of MPM registered annually in Australia, USA, Europe and Japan [14,15,16]. Data from the United States alone depict a steady rise in the incidence from 200 to 400 new cases per year in 2007 [17] (p. 1) to approximately 800 new cases per year in 2018 [11]. However, in a recent look at contemporary trends in MPM specific to the USA, Calthorpe et al. suggested that the increase in incidence was attributable to new diagnoses in women only, and when adjusting for age, the incidence over the last two decades has, in fact, remained stable [18]. Further to this, despite significantly more data available, there remain slight differences in the reporting of MPM epidemiology, with one review from 2018 suggesting that MPM occurs more frequently in females [2] (p. 538) and another review from 2022 suggesting an equal distribution between males and females [19]. It is proposed this heterogeneity stems from MPM being a rare malignancy.

Of the three different types of mesotheliomas, MPM constitutes up to 30 percent of all cases [13,17,18,19,20], with most of these cases occurring in Caucasians [13,21]. Cases of MPM have been reported within all age groups, with the overwhelming majority occurring in people over 50 years old [17,18,22] and less than five percent of the reported cases occurring in the first two decades of life [23]. MPM, however, does typically occur at a younger age than pleural mesothelioma, with an earlier median age at diagnosis [24].

In comparison to MPM, the development of pleural mesothelioma has a well-established association with asbestos, with more than 80 percent of cases in the literature having a link to prior exposure [2,24]. The link between MPM and asbestos exposure is not as strong, with only 30–50 percent of cases having an association [1,18,24,25]. The reported latency periods between exposure to asbestos and the development of different types of mesotheliomas also vary, with MPM averaging around 20 years, whilst the period for pleural mesothelioma is significantly longer, averaging around 30–40 years. Despite the lower number of MPM cases associated with prior exposure to asbestos, it remains a pertinent risk factor, evidenced by the guidelines from the PSOGI consensus meeting in 2018 recommending that anyone with prior exposure to asbestos undergo an informal screening program of annual abdominal imaging [9].

Mesothelioma oncogenesis is a complex interaction between multiple potential cofactors and somatic genetic events. Therefore, it is important to account for other risk factors associated with the development of MPM, despite some being extremely rare or even contentious in the literature. These include several germline mutations, such as the Breast Cancer (BRCA) gene, and genetic syndromes, such as BAP-1 tumour predisposition syndrome, Lynch syndrome, and Li-Fraumeni-like syndrome. Other risk factors include therapeutic irradiation; peritoneal irritation from previous surgeries; chronic peritonitis; autoimmune inflammatory processes affecting the abdomino-pelvic cavity such as Crohn’s disease; Hodgkin’s disease; endometriosis; exposure to erionite and thorotrast; talcum; some viruses including papovavirus and simian vacuolating virus; Mediterranean familial fever; and the presence of long-standing intra-abdominal catheters [2,19,24,25,26,27].

## 6. The Peritoneum and Pathogenesis of Malignant Peritoneal Mesothelioma

The peritoneum is the largest serous membrane in the human body, consisting of a single layer of mesothelial cells. It is composed of visceral and parietal components. The visceral peritoneum covers the intra-abdominal organs and mesentery and forms a continuous layer with the parietal peritoneum, which lines the abdominal wall and pelvic cavities [28]. The peritoneal cavity is the potential space between these two layers and contains a small amount of serous fluid. Acting as a large sac, intra-abdominal organs that are relatively fixed contain a mobile property to them due to their intimate relationship to the peritoneum. This mobility is aided by the hundreds of microvilli within the mesothelial cells, which also enable nutrient, waste, and gas exchange [29]. A dynamic organ, the peritoneum, plays a role in the exchange of peritoneal fluid, prevents fibrosis in the abdominal cavity and is in part responsible for the regulation of the body’s inflammatory responses by acting as a barrier to infection [30]. Any abnormality in these key functions leads to an imbalance in homeostasis and can precipitate disease processes, such as the overproduction of serous fluid resulting in ascites, the generation of fibrotic adhesions, inflammatory peritonitis and, in rare cases, primary malignancy. MPM is an example of this, arising from the mesothelial cells of the peritoneum.

Despite the weaker link between asbestos exposure and MPM than that of pleural mesothelioma, the proposed hypothesis for pathogenesis is similar. It is thought that exposure to the carcinogen causes the deposition of small fibre particles that damage DNA and consequently release oxygen free radicals, resulting in genomic instability [13]. The reactive oxygen and nitrogen species released by the mesothelial cells stimulate an inflammatory response by recruiting different molecules and proteins to enhance cell proliferation. Over time, this results in chronic inflammation, stimulating carcinogenesis of the mesothelial cells [30].

Recent molecular studies have proposed genetic mutations that may be related to the development of MPM in the absence of asbestos exposure. Comprehensive genomic analysis of malignant pleural mesothelioma has revealed loss of function alterations in tumour suppressor genes. BRCA-1-associated protein 1 (BAP1), Cyclin-Dependent Kinase inhibitor 2A (CDKN2A), and Neurofibromatosis type 2 (NF2) are the genes most frequently altered in pleural mesothelioma [31]. Consistent findings from genomic analysis of pleural mesothelioma have progressed the understanding of it being an immunogenic malignancy and, subsequently, pathways for immunotherapy clinical trials [32]. MPM has been found to have similar genomic alterations, however, the details are not as well established. Genetic alterations prevalent in both pleural and peritoneal mesothelioma include BAP1 and NF2 [13,33,34,35]. Loss of CDKN2A is present in MPM, however, less frequently than pleural mesothelioma cases [35]. Anaplastic Lymphoma Kinase (ALK) rearrangements have been found in MPM, whilst they are not present in pleural disease [35,36]. Overexpression of Programmed Death-Ligand 1 (PD-L1) has been identified in MPM, as well as multiple other mutations, including but not exclusively Ewing Sarcoma breakpoint region 1-Activating Transcription Factor 1 (EWSR1-ATF1), and FUS RNA binding protein (FUS) and ATF1 (FUS-ATF1) fusions [35,37,38]. Genomic studies find MPM to be underscored by genetic heterogeneity, with molecular differences between the various histological forms of mesothelioma, including diffuse, localised, and papillary. Given the rare nature of the disease, most genomic studies are analysed using single cases, and extensive further investigation is required to improve understanding and guide the future of targeted therapy for MPM [35].

## 7. Clinical Findings

Given MPM’s pattern of spread is more commonly of an expansive nature as opposed to infiltrative, symptoms are usually associated with the extent of tumour load within the abdominal cavity. Presenting symptoms can vary, but in up to 50 percent of patients, they usually include generalised and non-specific abdominal pain, abdominal distension, and bloating, anorexia, weight loss, early satiety, and altered bowel habits [2]. Of note, the abdominal distension is usually attributable to large-volume ascites, and occasionally patients may present with a palpable abdominal mass or intestinal obstruction [39]. Another common mode of presentation is by incidental finding, either on cross-sectional imaging or during an intra-abdominal procedure for other abdominal pathology [9]. The non-specific clinical findings of MPM contribute to delayed diagnosis, and it has been reported that the average time of symptom onset to formal diagnosis ranges from four to six months but could often be longer [19,40]. Often, by this stage in the disease progression, patients will have diffuse disease throughout the abdomen [39].

## 8. Diagnosis

MPM diagnosis is reached by a combination of history, physical examination, laboratory assessment, radiological investigation and sometimes diagnostic laparoscopy. The final pathological diagnosis is made from the assessment and evaluation of multiple core biopsies. As already mentioned, there are several patients who are diagnosed with MPM by incidental finding, however, for the patients that present with suspicious features of MPM on history and examination, cross-sectional imaging with computed tomography (CT) of the abdomen and pelvis will be undertaken. This imaging modality is widely accepted as the preferred radiological investigation in a diagnosis of MPM [9,24].

## 9. Imaging

**Figure 1:**
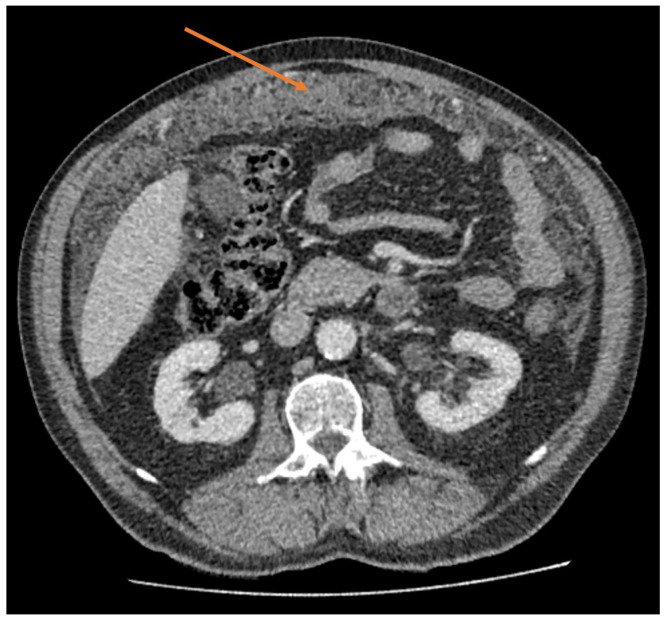
Abdominal CT scan (axial view) demonstrating significant diffuse thickening of the greater omentum (orange arrow), also known as “omental caking”.

Features of MPM on CT include a solid, soft tissue mass with a heterogenous appearance and irregular margins, which enhances in the presence of intravenous contrast [2]. A diffuse distribution within the abdominal cavity is common on CT, owing to the expansive nature of the spread. In addition to the mesenteric or parietal peritoneal nodules, other features that typically appear on CT include visceral peritoneal thickening that leads to the foreshortening of the mesentery with an uncharacteristically straight course of the vessels, ascites, and thickening or caking of the omentum, as seen in Figure 1 [2,19]. Possible differentiating features of MPM from other malignancies on CT include the lack of a distinct primary site of malignancy, lymph node involvement, or extra-abdominal metastases [39].

Presentations of MPM on CT can, however, vary, and a classification system has been used to elucidate this. The dry-painful type describes abdominal pain associated with peritoneum-based masses; the wet type describes abdominal distension associated with ascites and multiple nodules; and the mixed type is a combination of the two [41]. A CT scoring system for small bowel and mesentery involvement was described by Yan et al. to evaluate the resectability of the disease [42]. Analysing results of patients who had cytoreduction surgery with peri-operative intraperitoneal chemotherapy, the authors identified radiological features, including a tumour mass of more than 5 cm in the epigastric region and loss of normal architecture of the small bowel as key unfavourable prognostic anatomical findings on CT [42].

There are limited data regarding the use of Magnetic resonance imaging (MRI) and Positron-emission tomography (PET) in the diagnosis of MPM, and there is no clear evidence to date that either modality is more effective than CT in assessing small bowel involvement in MPM. Both modalities, however, have demonstrated some additional benefits. It has been reported that in the presence of high-volume ascites, MRI may be more effective than CT in evaluating tumour involvement [43]. In another study, MRI was reported as superior to CT in calculating the radiological peritoneal carcinomatosis index (PCI) [44]. Furthermore, the use of Fluorine-18 fluorodeoxyglucose (18-FDG)-PET in the workup of MPM patients has recently been described, with both sensitivity and specificity above 85 percent [45]. Both MRI and PET can provide precise staging information and aid in pre-operative planning, however, despite the reported advantages of using these imaging modalities, there is a scarcity of data in the literature regarding their use for MPM specifically. It, therefore, remains an area of study that requires further investigation in the future.

## 10. Tumour Markers

The use of tumour markers in the diagnosis of MPM has been reported in the literature [9,24,27,46]. Serum protein cancer antigen (CA)-125 and CA15-3 are frequently raised in those patients diagnosed with MPM, however, reported sensitivities are relatively low at 53 and 48.5 percent for CA-125 and CA15-3, respectively [46]. In a study evaluating the use of tumour markers in MPM, out of 22 patients who had a pre-operatively raised CA-125 and went on to have cytoreductive surgery with adequate disease clearance, 21 of them had a negative CA-125 in their post-operative surveillance tests [47]. Other serum tumour markers that have been suggested as useful include osteopontin and mesothelin-related protein (SMRP) [48]. Ultimately, with varying degrees of efficacy in the literature for tumour markers as a diagnostic tool in MPM, their current role in clinical practice is less diagnostic as it is for monitoring treatment response, recurrence and surveillance.

## 11. Tissue, Cytology and Immunohistochemistry

Confirmation of diagnosis is made by assessment of tissue specimens obtained either radiologically by percutaneous core needle biopsy or surgically by laparoscopic or open intra-abdominal direct tissue sampling. Given one of the most common clinical findings in MPM includes ascites, in the past, it was routine to perform cytological assessment of this fluid. However, more recently, fluid cytology alone has been shown to have low diagnostic yield for MPM, as the low number of malignant cells in the fluid is often inconclusive [11,24,27,39]. The PSOGI/EURACAN Clinical Practice Guidelines addressed this and mandated that definitive diagnosis of MPM requires histopathological analysis of adequate tissue specimens rather than cytological examination, and in addition, that each specimen be reviewed by a pathologist with expertise in peritoneal surface malignancies [9].

Immunohistochemical (IHC) staining is critical for the definitive diagnosis of MPM in order to differentiate it from other intra-abdominal malignancies with similar cellular profiles. Current guidelines recommend the use of at least two mesothelioma and two carcinoma markers [39]. Positive stains for MPM include calretinin, Wilm’s tumour (WT-1) and cytokeratin 5/6, which are the most sensitive, as well as mesothelin, osteopontin, podoplanin (D2-40), fibulin-3, vimetin, human mesothelial cell (HBME-1) and epithelial membrane antigen [21,25,49,50]. Negative stains include carcinoembryonic antigen (CEA), Ber-Ep4, LeuM1, Bg8, Pax-2, B72.3, and thyroid transcription factor-1, which all aid in differentiating MPM from other malignancies, such as adenocarcinoma [15,25,49,50]. In addition, it has recently been demonstrated that the loss of nuclear BAP1 on IHC is an accurate marker of mesothelioma with high sensitivity and specificity and can be used to confirm the diagnosis of MPM [21,49].

## 12. Staging

Laparoscopy has been used as part of a diagnostic as well as a staging tool for peritoneal surface malignancies. Generally, it has been demonstrated as a safe, feasible, and valuable addition to the workup of a patient with peritoneal carcinomatosis [51,52,53]. Specific to MPM, however, there is only one study in the literature that evaluates laparoscopy [54]. In this study, the authors assessed the reliability of exploratory laparoscopy in selecting patients that were potential candidates for combined cytoreductive surgery and hyperthermic intraperitoneal chemotherapy (HIPEC) in predicting complete cytoreduction. Out of 33 patients who had a diagnostic laparoscopy, 30 were deemed amenable to complete cytoreduction. Out of these 30 patients, the authors reported complete cytoreduction in 29 of them, suggesting the conclusions reported for diagnostic laparoscopy in peritoneal surface malignancies could be extrapolated to MPM. The advantages of diagnostic and staging laparoscopy in MPM include direct visualisation of the abdomen, allowing for PCI scoring and potential pre-operative planning, the ability to obtain tissue biopsies, and the possible benefit of providing further information in those patients whose radiological findings may not have been conclusive. Possible disadvantages of diagnostic laparoscopy include the potential to underestimate the tumour burden as well as port site tumour seeding. Therefore, if diagnostic and staging laparoscopy is performed, the number of ports used should be limited and ideally placed in the linea alba [11,19].

**Figure 2:**
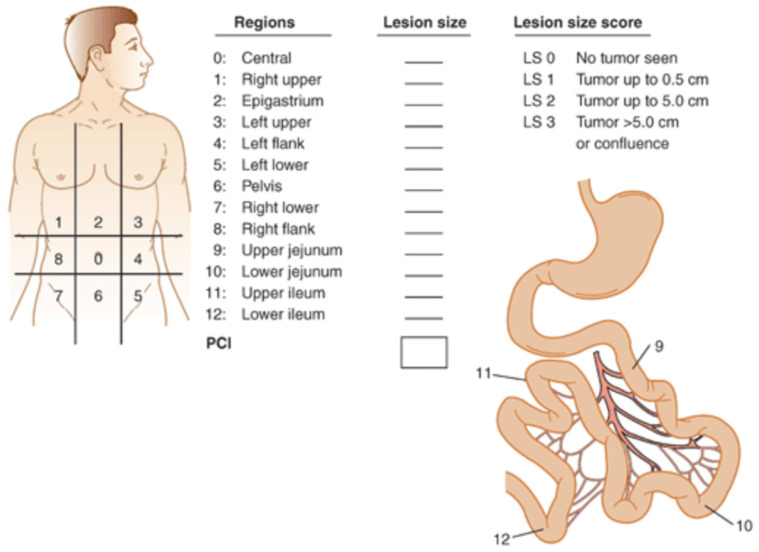
Peritoneal carcinoma index score [57].

The PCI score is universal in quantifying peritoneal disease burden and is used for staging and prognostic purposes. This scoring system divides the abdomen into nine separate regions by two transverse and sagittal planes, each as seen in Figure 2. Each region is allocated a number from zero to eight, and in addition to this, the small bowel is divided into four separate components according to upper or lower jejunum and ileum, numbered nine to twelve. A lesion score is assigned to each region based on the amount of macroscopic disease ranging from zero to three, and the total score for all thirteen regions is the PCI. The lowest PCI is zero, and the highest is 39. Given that MPM typically spreads diffusely rather than in an infiltrative pattern, haematogenous and lymphatic metastatic potential remain relatively low [55]. The common TNM staging system is, therefore, somewhat incongruous and less appropriate for use in MPM. Yan et al., in 2011, developed a novel TNM staging system specific to MPM in order to address this [56]. The authors proposed that T is assigned to the extent of peritoneal disease burden, N to intra-abdominal nodal metastases and M to extra-abdominal metastases. According to this staging system, 5-year survival ranged from 87% for stage I to 29% for stage III [56].

## 13. Histopathological Subtypes

Peritoneal mesothelioma has been described as a somewhat orphan disease, as previously, most of the knowledge relating to its pathology and natural history was extrapolated from publications focusing on pleural mesothelioma. However, in a recent article, Chapel et al. suggested that several pathological parameters observed in pleural mesothelioma are transferrable to peritoneal mesothelioma with similar results [58]. In addition, the last two decades have witnessed large growth in the number of publications relating to the diagnosis and pathological classification of peritoneal mesothelioma specifically.

Peritoneal mesothelioma represents a spectrum of primary peritoneal tumours with varying degrees of malignant biology and clinicopathological behaviours. This spectrum ranges from benign tumours to more aggressive papillary subtypes. Multicystic mesothelioma and well differentiated papillary mesothelioma (WDPM) are at the lower end of the spectrum, representing borderline malignancies classified as low-grade tumours [24]. These variants are considered separate biological entities from the malignant tumours and are usually differentiated through their lack of invasion, as well as their minimal to no increase in stromal cellularity [19]. However, despite their indolent behaviour, there are cases where both multicystic mesothelioma and WDPM have been reported to transform into more malignant subtypes [59].

On the other end of the scale is the more aggressive papillary type known as diffuse malignant peritoneal mesothelioma (DMPM), commonly referred to as MPM throughout the literature. MPM is divided into three histopathological subtypes: epithelioid, sarcomatoid and biphasic/mixed. Multi-institutional registry data reported that of these, the most common type is epithelioid, accounting for approximately 80 percent of MPM, whilst the remainder of cases is accounted for by the less common subtypes biphasic (approximately 13 percent) and sarcomatoid (which is rare) [14,15]. Out of the three, epithelioid is the least aggressive tumour and is associated with the most favourable outcomes, whereas sarcomatoid and biphasic are considered highly aggressive with rapid local progression, more infiltrative growth patterns and poorer outcomes [24]. Histologically, epithelioid resembles normal mesothelial cells in a tubulo-papillary pattern with rare mitotic figures [39]. In epithelioid tumours, cellular atypia is a frequent occurrence but is typically mild [17]. Sarcomatoid, on the other hand, is characterised by a fascicular proliferation of tightly packed spindle cells and demonstrates significantly more cellular atypia [17]. The biphasic subtype includes both epithelioid and sarcomatoid cellular characteristics.

## 14. Treatment

### 14.1. Chemotherapy

Traditionally, MPM was considered an untreatable disease, and management was predominantly best supportive care comprising of paracentesis and palliative systemic chemotherapy regimens adopted from experience with pleural mesothelioma. Despite the tumour biology between pleural and peritoneal mesotheliomas demonstrating significant differences, the efficacy of systemic chemotherapy agents is reportedly similar in both diseases [60]. A phase III randomised trial in 2003 reported improved survival when using a combination of pemetrexed and cisplatin versus cisplatin alone and was, therefore, the impetus for universal acceptance of pemetrexed combined with a platinum-derivative as first-line therapy in patients having systemic chemotherapy for MPM [61]. As a second-line option, the combination of pemetrexed and gemcitabine has been recommended in patients who cannot tolerate platinum therapy [9]. However, the use of either single- or dual-agent systemic chemotherapy has demonstrated little survival benefit in MPM, as both are associated with poor response rates of less than 20 percent and median survival of approximately one year [61,62]. Therefore, systemic chemotherapy alone is typically reserved for those patients with unresectable disease, deemed unfit for operative intervention or those who choose not to undergo an operation [29,63].

### 14.2. Peri-Operative Chemotherapy

The effect of systemic chemotherapy in the peri-operative setting for patients who undergo CRS and HIPEC is still widely unknown. A multi-centre, retrospective study from 2016 of 126 patients who had CRS and HIPEC reported that adjuvant chemotherapy may delay recurrence and improve survival, however neoadjuvant chemotherapy may negatively impact survival in patients undergoing CRS and HIPEC for MPM with curative intent [64]. More recently, in another retrospective analysis of patients having surgery for MPM with curative intent, the authors suggested that whether systemic chemotherapy was given before or after CRS and HIPEC, the addition was associated with survival improvement at one year, however, this benefit did not extend beyond 12 months [65]. Despite contrasting results, both studies suggested that CRS and HIPEC remain the standard treatment in appropriately selected candidates. A recent recommendation from the PSOGI/EURACAN clinical practice guidelines addressed adjuvant chemotherapy, suggesting that after CRS and HIPEC, patients who have at least one poor prognostic factor out of CC-score more than one, sarcomatoid or biphasic histology subtype, lymph node involvement, Ki67 more than nine percent or PCI more than seventeen, should be considered for combined systemic chemotherapy rather than surveillance alone [9].

### 14.3. Cytoreductive Surgery

CRS and HIPEC evolved into mainstay treatment for peritoneal surface malignancies in the 1990s, and after two international consensus meetings in 2004 and 2006, it was established as the standard of care for MPM in appropriately selected patients [7,8]. However, appropriate patient selection for surgery is critical given the extensive nature of this treatment modality. The decision to offer CRS and HIPEC to patients with MPM is based on their age, performance status, histological subtype of disease and the predicted ability to achieve complete cytoreduction during surgery [2,55]. Favourable patient factors include age less than 60, Eastern Cooperative Oncology Group (ECOG) score of zero or one, female sex, epithelioid histopathologic subtype and radiologic PCI favouring complete cytoreduction [21]. The ability to achieve complete cytoreduction is prioritised as it is the main determinant of outcome after surgery [24]. Therefore, pre-operative evaluation of some specific radiographic criteria is paramount in making the decision whether to operate or not, as findings of segmental obstruction of the small bowel, tumour nodules more than five centimetres, or those located directly adjacent to the mesentery of the jejunum or ileum all predict less chance of complete cytoreduction [42]. Large tumour mass in the lesser omentum, extra-abdominal disease and para-aortic lymph node metastases are also associated with poor outcomes and should, therefore, be considered in the decision-making process [60]. In addition, severe cardiac, pulmonary, hepatic or renal dysfunction are also contraindications as they correlate to poor performance status [66]. Interestingly, another factor that should be considered is pre-operative thrombocytosis, as one retrospective review reported poorer median survival compared to those patients who had normal platelets prior to surgery [67]. Ultimately, the treatment algorithm for patients with MPM is complex, and the decision to operate on these patients should only be made at a multidisciplinary team (MDT) meeting, and surgical intervention should be undertaken only by an experienced and dedicated peritoneal surface malignancy surgical unit.

The diffuse and expansive nature of disease within the peritoneal cavity in patients with MPM usually requires an extensive peritonectomy procedure to achieve complete cytoreduction. The extent of cytoreductive surgical strategy varies according to institutional preference, with no clear guidelines as to which one is superior. Selective peritonectomy is a variant of the operation that comprises peritonectomy only to peritoneal surfaces visibly infiltrated by disease, whereas systematic peritonectomy is made up of a total parietal peritonectomy [68]. Proponents of the selective peritonectomy suggest that the parietal peritoneum makes up less than 20 percent of the total peritoneal surface area and, therefore, a more aggressive approach to resecting parietal peritoneum that is macroscopically normal may not be valuable. Those in favour of complete parietal peritonectomy argue that the heterogeneity in peritoneal biology renders some areas of the parietal peritoneum more susceptible than others to neoplastic implants and potential microscopic disease, which could negatively impact prognosis [9]. Baratti et al. reported the risk of microscopic involvement of macroscopically appearing normal parietal peritoneum may be more than fifty percent [68]. In addition, the authors reported an improved five-year survival with the total parietal peritonectomy over the selective procedure, with no significant difference in morbidity or mortality [68]. Despite differences in opinion and approach, the systematic peritonectomy procedures in CRS for MPM will include greater omentectomy-splenectomy, lesser omentectomy-cholecystectomy with stripping of the omental bursa, left and right upper quadrant peritonectomy and pelvic peritonectomy with sleeve resection of the rectosigmoid colon if indicated [21].

### 14.4. HIPEC

After CRS, if complete or near-complete cytoreduction has been achieved, HIPEC should be administered in order to cause a cytotoxic effect to the microscopic tumour cells. The hyperthermic effect is directly cytotoxic by impairing DNA repair, denaturing proteins and inducing heat-shock proteins, which will result in apoptosis and inhibition of angiogenesis [60]. Hyperthermia also works synergistically with the cytotoxic agent infused into the peritoneal cavity to increase potency. There are no guidelines as to the gold standard of HIPEC agent to use, and therefore, there is institutional variability within the literature. Standard recommendations usually comprise either the use of cisplatin supplemented by doxorubicin or mitomycin C or a combination [11,21]. A retrospective study from 2018 evaluated survival outcomes using different HIPEC agents and reported no statistical difference between the different agents, however, survival improved when using two agents compared to one [69]. An important consideration in administering HIPEC to patients with MPM is that, unlike other peritoneal surface malignancies, if a patient with MPM has an incomplete cytoreduction but a significant debulking, HIPEC has been associated with improved survival and should, therefore, be factored into their procedure [11].

Additional methods of intraperitoneal chemotherapy have been reported. Early post-operative intraperitoneal chemotherapy (EPIC) comprises the administration of chemotherapeutic agents from post-operative day one to five through an abdominal inflow and outflow drain inserted during the initial procedure. There are some reports that EPIC may be beneficial, however, disadvantages may include an increased risk of infection and an array of increased post-operative complications [21,60]. Another option considered by some institutions is normothermic intraperitoneal chemotherapy long-term (NIPEC-LT). Administered through an intraperitoneal port placed at the time of the initial surgery, NIPEC, in addition to CRS and HIPEC, as well as systemic chemotherapy, has demonstrated a significantly improved overall survival when compared to CRS and HIPEC alone or CRS and HIPEC plus EPIC [70].

The evolution of cytoreductive surgery (CRS) and heated intraperitoneal chemotherapy (HIPEC) in appropriately selected patients with MPM has seen a significant improvement in survival. Yan et al. published a review of 401 patients treated with at least CRS and HIPEC, reporting a five-year overall survival of 47 percent and an overall median survival of 53 months [14]. In 2015, Helm et al. published the largest review of CRS and HIPEC for MPM at that time with a systematic review and meta-analysis, demonstrating improved outcomes versus historical controls [1]. The most recent study evaluating outcomes in patients with MPM having CRS and HIPEC is a multi-institutional retrospective analysis of 72 patients treated at four major peritoneal disease centres in Canada between 2000 and 2021. The authors reported a five-year overall survival of 61% [71] (page. 4), providing further evidence of improved survival outcomes in patients with MPM treated with CRS and HIPEC at specialty centres. Similarly, another recent retrospective cohort analysis of 1998 patients with MPM spanning a period of over 40 years from the SEER database showed that patients receiving surgical intervention had a better five-year survival versus chemotherapy or radiotherapy alone [13]. In this same study, however, the authors reported that more than 55 percent of patients received chemotherapy alone, only 29 percent were treated with surgery, less than two percent of patients had radiotherapy alone, and a staggering 18.7 percent of patients did not have any treatment data recorded [13]. In addition, another study demonstrated that diagnosis from 2015 to 2018 was associated with improved survival when compared to diagnosis between 2000 and 2002, with the authors postulating that factors such as increased universal adoption of CRS and HIPEC, as well as optimisation of the actual chemotherapeutic agents in HIPEC were accountable for the increased survival data [18].

### 14.5. Repeat CRS

Despite improved outcomes in patients with MPM having CRS and HIPEC, given its aggressive tumour biology, disease recurrence can occur and represents a therapeutic challenge. A retrospective cohort analysis of a prospectively maintained database from 1989 to 2012 asserted that re-do CRS and HIPEC for MPM in appropriately selected patients was safe and efficacious with an improved median survival [72]. However, incomplete cytoreduction was significantly more frequent in the repeat CRS group, therefore demonstrating the need to instil rigorous patient selection methods in those with recurrent disease.

## 15. Morbidity and Mortality

CRS and HIPEC can result in significant morbidity and mortality. The learning curve to develop a level of expertise is steep, with data suggesting that 140 procedures are required to reach this level [73]. Another study reported that at least 100 procedures were required to reach proficiency and suggested that the most important institutional factor impacting performance was the volume of cases [74]. In 2009, data from the largest peritonectomy unit in the Southern Hemisphere reported on the outcomes of 20 patients [75]. Four patients had a major morbidity (defined as Clavien-Dindo III or above), and there was one mortality after fourteen days. The same unit published updated data in 2022 on 81 patients treated for MPM from 1999 to 2021, reporting that morbidity and mortality remained acceptable at 43.2 percent and under five percent, respectively [76]. These numbers are similar to those published by Yan et al. in a systematic review of seven studies from six specialist CRS centres, reporting a range of morbidity from 25 to 40 percent, and a range for mortality from zero to eight percent [77]. In their multi-institutional retrospective analysis, Deban et al. reported Clavien-Dindo grade III and IV complications of ten and seven percent, respectively, attributing their lower rates to appropriate patient selection and expertise in CRS and HIPEC across the four centres [71].

## 16. Novel Therapies

There is a paucity of data investigating molecular and immune therapy in MPM. Despite most of the research in this area aimed at pleural mesothelioma, there is promising research demonstrating molecular pathways that can be manipulated in the treatment of MPM [2,55]. A potential target established for personalised therapy is the ALK status in patients with MPM. Functional and clinically actionable genetic rearrangements were recently identified in three percent of patients, whilst this rearrangement was not demonstrated in pleural mesothelioma [36]. A recent study identified that there are only four cases reported of patients with MPM who have an ALK translocation with STRN as the fusion partner [78]. In this case report, the authors presented a seventeen-year-old female patient with no history of asbestos exposure who had MPM with STRN-ALK gene fusion. Tumour progression was observed with standard chemotherapy, and the patient was commenced on alectinib, a Tyrosine Kinase receptor inhibitor, as part of a clinical trial that has not yet been published [78]. Another recent trial on alectinib which has been published investigates its use for unresectable malignancies, with the authors reporting it may be effective in ALK-positive cancers [79]. Erlotinib and gefitinib are first-generation tyrosine-kinase inhibitors aimed at targeting the epidermal growth factor receptor (EGFR), which is over-expressed more in MPM than pleural mesothelioma. Despite this, these agents are still yet to show any significant activity in any type of malignant mesothelioma [80]. More promising results have been reported in an investigation of an angiokinase inhibitor aimed at vascular endothelial growth factor receptors (VEGF), with progression-free survival improving in patients with malignant pleural mesothelioma treated with nintedanib in combination with systemic chemotherapy [81]. Another anti-vascular endothelial factor agent, bevacizumab, in addition to systemic chemotherapy, was reported to have significantly improved overall survival in patients with unresectable pleural mesothelioma [82]. Similarly, tremelimumab, a monoclonal antibody targeting the cytotoxic T-lymphocyte antigen-4 (CTLA4), demonstrated efficacy as a second-line regimen in malignant pleural mesothelioma and MPM [83]. Another monoclonal antibody targeted therapy is the immune checkpoint inhibitor nivolumab, which is a Programmed Death-1 (PD-1) inhibitor. A recent report on a 70-year-old male with MPM refractory to chemotherapy described the use of Nivolumab as a salvage therapy for this patient [84]. The authors reported a positive clinical response with symptom reduction, providing further evidence for the possible utility of immunotherapy in MPM. One of the most recent studies of immunotherapy in MPM was published in 2023, which reviewed the use of single or dual-agent immunotherapy or immunotherapy combined with chemotherapy. The authors concluded that due to the small number of patients with MPM in the clinical trials performed to date, no definitive conclusions could be reached, and therefore, no firm recommendations were made [85]. The authors proposed that given the lack of clear evidence regarding immune checkpoint inhibitors in comparison to chemotherapy as a first-line therapeutic choice for MPM, it would be reasonable to reserve them for use as a later option [85]. The use and outcome of molecular and immune therapy in MPM remain vastly unknown and are an extremely promising area of investigation for the future.

## 17. Conclusions and Future Developments

MPM is a rare primary tumour of the intra-abdominal serosal membranes. There are three main histological subtypes, with sarcomatoid and biphasic being associated with significantly worse outcomes than epithelioid. Historically, this aggressive tumour would render patients untreatable with a dismal prognosis. Initially, palliative systemic chemotherapy was the only treatment option, however, in recent decades, CRS and HIPEC have emerged as the gold standard of treatment in appropriately selected patients, with an overwhelming number of published data demonstrating improved overall survival. Completeness of cytoreduction remains the single most important prognostic factor, however, other patient factors impacting selection for CRS and HIPEC include age, performance status, histological subtype, and pre-operative PCI. Given the pattern of spread in MPM is usually diffuse and expansive rather than infiltrative, it is usually restricted to the abdominal cavity and extra-abdominal metastases are uncommon. Therefore, chemotherapeutic agents infused into the abdominal cavity, either during CRS, in the days immediately after, or over a longer period in the post-operative phase, have demonstrated improved overall and median survival. More recently, molecular characterisation of malignant mesothelioma has improved treatment options for patients with pleural mesothelioma. Currently, phase II and III trials are aimed at identifying novel molecular targets in MPM, specifically with the objective of offering potential new treatments and subsequently improved outcomes for patients.

## Data Availability

No new data were created or analyzed in this study. Data sharing is not applicable to this article.
